# Complete mitochondrial genome sequence of *Emberiza sulphurata* (Emberizidae: *Emberiza*)

**DOI:** 10.1080/23802359.2017.1289351

**Published:** 2017-02-16

**Authors:** Mu-Yeong Lee, Hey Sook Jeon, Junghwa An

**Affiliations:** Animal Resources Division, National Institute of Biological Resources, Incheon, Republic of Korea

**Keywords:** *Emberiza sulphurata*, genome, mitochondrion, Japanese yellow bunting

## Abstract

The Japanese yellow bunting (*Emberiza sulphurata*) is considered to be an endangered species. Here, the complete mitochondrial genome of *E. sulphurata* (16,797 bp in length) was determined. The genome consists of 37 genes (13 protein-coding genes, two ribosomal RNA genes, and 22 transfer RNA genes) and one control region (D-loop), and so is highly similar in architecture to the typical vertebrate mtDNA genome. The base composition of the mtDNA was A (29.9%), C (32.6%), G (14.5%), and T (23.0%), so the percentage of A and T (52.9%) was slightly higher than that of G and C. All the genes in *E. sulphurata* were encoded on the H-strand, except for the genes for the *ND6* subunit and eight tRNAs, which were encoded on the L-stand. Phylogenetic analysis using Emberizidae mitogenomes revealed that *E. sulphurata* was grouped into the family Emberizidae and that *E. spodocephala* is the most closely related species.

The Japanese yellow bunting, *Emberiza sulphurata* (Passeriformes, Emberizidae, *Emberiza*), is a passerine bird, found in eastern Asia. The yellow bunting breeds only in Japan but principally winters further south in China, Taiwan, and the Philippines. In South Korea, it is a migratory bird, visiting in spring and fall (NIBR 2011). *Emberiza sulphurata* populations are small and have undergone a significant decline in number during the course of the twentieth century. The cause of the population decline is probably a combination of a number of factors, including habitat loss, pesticide contamination, and trapping for the caged-bird trade (BirdLife International 2016). *Emberiza sulphurata* is listed as a Class II endangered species by the Korean Ministry of Environment and is also listed as vulnerable on the International Union for the Conservation of Nature (IUCN) red list.

Mitochondrial DNA (mtDNA), is an important molecular marker, and is commonly used to study evolutionary relationships between species as well as the evolutionary history of a population (Irwin et al. 1991). In addition, it has frequently been applied in conservation genetics including phylogeography (Avise 2000). In this regard, many complete *Emberiza* mitochondrial genome sequences have been reported (Kan et al. 2013; Pan et al. 2013; Chen et al. 2014; Hu et al. 2014; Ren et al. 2014a, 2014b; Pan et al. 2015a, 2015b; Sun et al. 2016; Zhao et al. 2016).

The yellow bunting used in this study was obtained from a location in Sinan-gun, Jellanamdo, South Korea. Total genomic DNA was extracted from a muscle tissue sample using a DNeasy Blood & Tissue Kit (Qiagen, Valencia, CA) following to the manufacture’s protocol. The complete mtDNA of *E. sulphurata* (Accession number KY419885) was amplified and sequenced using 20 pairs of PCR primers.

The total length of the *E. sulphurata* genome is 16,797 bp, consisting of 13 protein-coding genes, 22 transfer RNA genes, 2 ribosomal RNA genes, and one control region (D-loop region). A tandem repeat was not found in the D-loop region. As in most vertebrates, all of the genes in *E. sulphurata* were encoded on the H-strand, except for the genes encoding the *ND6* subunit and eight tRNAs, which were encoded on the L-stand. The overall base composition of the mtDNA was A (29.9%), C (32.6%), G (14.5%), and T (23.0%), with the percentage of A and T (52.9%) being slightly higher than that of G and C (47.1%).

Eleven Emberizidae mitogenomes including the *E. sulphurata* mitogenome generated in this study were used to construct a phylogenetic tree based on a concatenation of the sequences of the 13 protein-coding genes (11.398 bp) with out-group taxa. A neighbor-joining analysis demonstrated that the species of Emberizidae in this study form a monophylogenetic clade and that *E. sulphurata* is closely related to *E. spodocephala* (Figure 1). It is anticipated that the complete mitogenome of *E sulphurata* will provide key data for further phylogenetic analyses of Genus *Emberiza* species.

**Figure 1.**
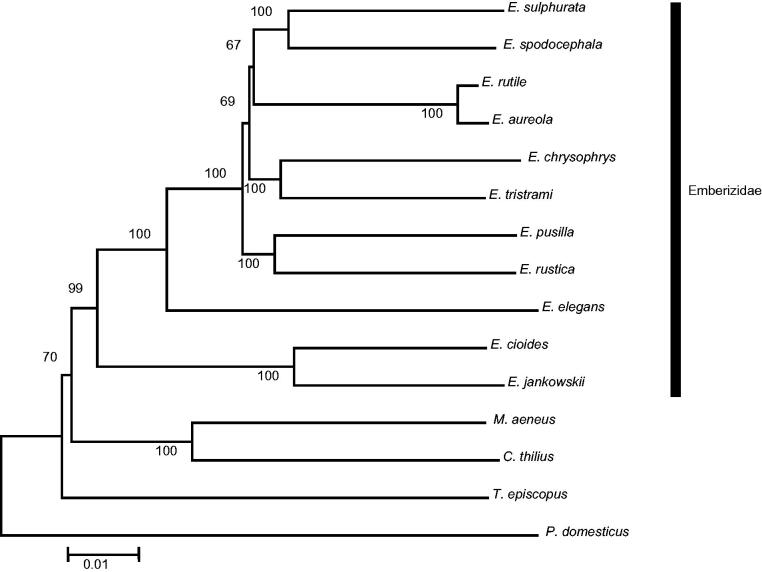
A phylogenetic tree of 11 Emberizidae species based on K2P parameters constructed using the Neighbour-Joining algorithm using MEGA 6.0 software. Bootstrap replicates were performed 1000 times. GenBank accession numbers: *Emberiza aureola*, KF111713; *E. cioides*, KF322027; *E. chrysophrys*, HQ896034; *E. jankowskii*, KP738714; *E. pusilla*, KC407232; *E. rustica*, KC831775; *E. rutile*, KC952874; *E. spodocephala*, KC758647; *E*. *sulphurata*, this study; *E. tristrami*, HQ896035; *Chrysomus thilius*, JX516069; *Molothrus aeneus*, JX516067; *Passer domesticus*, KM078784; *Tangara episcopus*, KM078765.
